# Verification of using virtual reality to evaluate deficiencies in cognitive function among patients with schizophrenia in the remission stage: a cross-sectional study

**DOI:** 10.1186/s12888-020-03029-6

**Published:** 2021-01-09

**Authors:** Bochao Huang, Shangda Li, Bing Sun, Hailong lyu, Weijuan Xu, Jianping Jiao, Fen Pan, Jianbo Hu, Jinkai Chen, Yaping Chen, Manli Huang, Yi Xu

**Affiliations:** 1 Department of Psychiatry, The First Affiliated Hospital, Zhejiang University School of Medicine, The Key Laboratory of Mental Disorder’s Management of Zhejiang Province, Zhejiang University Brain Research Institute, Zhejiang Engineering Center for Mathematical Mental Health, 79 Qing Chun Road, Hangzhou, 310003 Zhejiang Province China; 2 Ningbo Psychiatric Hospital, 11 Rixingfang, Jiangbei District, Ningbo, 315032 Zhejiang province China

**Keywords:** Virtual reality, Schizophrenia, MCCB, Cognitive function

## Abstract

**Background:**

Schizophrenia is associated with widespread cognitive impairment. The MATRICS Consensus Cognitive Battery (MCCB) is most frequently used to assess cognitive function. However, the MCCB test is time consuming for the clinician. Virtual reality (VR) has emerged as an adjunctive tool to overcome this limitation and provides a new means to assess cognitive function.

**Methods:**

The present study examined the validity and safety of using VR technology to assess cognitive function in Han Chinese patients with schizophrenia (SZs). The VR cognition training system (VRCTS) was used to simulate real-life supermarkets and assess cognitive function. Thirty-two SZs and 25 healthy controls (HCs) underwent VRCTS and MCCB assessments. An auxiliary diagnosis model was created based on the outcomes of the VRCTS to classify SZs and HCs by cognitive impairment.

**Results:**

Significant differences in completion time between the SZs and HCs were detected using the VRCTS. SZs spent more time completing tasks than HCs. The outcome of VRCTS significantly correlated with the MCCB. The auxiliary diagnosis model had a sensitivity of 88.89% and a specificity of 88.89%.

**Conclusions:**

These results support the use of VR technology in the assessment of cognitive impairment in Han Chinese schizophrenia patients.

**Trial registration:**

China Clinical Trial Registry, ChiVTR1800016121. Registered 13 May 2018, http://www.chictr.org.cn/showproj.aspx?proj=27233

## Background

Schizophrenia is a complex, heterogeneous behavioural and cognitive syndrome that is characterized by positive symptoms, negative symptoms and cognitive impairment [1, 2]. Patients with schizophrenia (SZs) exhibit diminished cognitive function, including reduced attention and memory, and difficulties with executive functioning [3]. The MATRICS Consensus Cognitive Battery (MCCB), which includes 10 different cognitive subtests, is an accepted standard for the measurement of cognitive change in schizophrenia, and it is recommended by the United States Food and Drug Administration (FDA) for the assessment of cognitive impairment in schizophrenia [4, 5]. The MCCB demonstrates excellent reliability and practicality. Recent studies showed that the MCCB was applicable for first-episode schizophrenia and chronic schizophrenia [6], and investigations using the MCCB focused on different cognitive domains in SZs. One study showed that the parents of SZs have conspicuous dysfunction in domains of working memory (WM), problem reasoning and visual learning (ViL) compared to parents of healthy controls (HCs) [7]. However, the MCCB requires a well-trained psychiatrist, and it takes approximately 1 h to evaluate cognitive function. The MCCB is also complicated for some SZs. Some patients may feel bored and exhausted to the point where they cannot complete the assessment. Therefore, it is necessary to find an easier and more attractive method to evaluate cognitive function.

Virtual reality (VR) has emerged as a tool to overcome this limitation of the MCCB and provide a new means to assess cognitive function. VR is a powerful tool that creates interactive computer-generated worlds that produce the sensation of being present in life-sized environments [8]. VR is already used in psychiatry. Current evidence suggests that the use of VR in SZs has great advantages. Some previous studies focused on the assessment of cognitive function using VR technology. A VR navigation task (VRNT) study reported that SZs showed significant impairment in memory compared to normal subjects [9]. Thirty-nine SZs and 21 HCs experienced a virtual maze, and SZs exhibited a higher rate of error [10]. Another study showed that SZs were deficient in life activities, medication management skills, and virtual character recognition [11–13].

Although studies reported the application of VR in psychiatry, there is limited literature on VR applications in Han Chinese people. A study in Hong Kong showed that VR provided a sensitive assessment of prospective memory deficits in SZs [14], but the efficiency and safety of VR technology are not sufficiently clear. The association between the VR method and the MCCB in the assessment of the cognitive function of SZs lacks evidence. Two studies in the United States demonstrated that the completion time of a VR task to assess functional capacity correlated with MCCB composite scores [15, 16].

The present study examined VR technology performance for the assessment of cognitive function and the reliability of VR in distinguishing HCs and SZs in the remission stage in people of Han Chinese descent by cognitive function.

## Methods

### Participants

35 SZs in the Ningbo Psychiatric Hospital were recruited under the supervision of Dr. Sun, and 25 HC volunteers were recruited from the general population. All of the participants entered the VR environment, named the VR cognition training system (VRCTS), and underwent the MCCB test. The outcomes of VRCTS in the two groups were compared, and an auxiliary diagnosis model was created based on the outcomes of VRCTS to classify SZs and HCs using a support vector machine (SVM) method. Another 9 SZs and 9 HCs were recruited for verification of the auxiliary diagnosis model.

The following inclusion criteria were used: aged between 18 and 55 years; met the ICD-10 criteria for schizophrenia; Positive and Negative Syndrome Scale item scores of ≤3 or SAPS and SANS item scores of ≤2 for at least 6 months according to Andreasen’s criteria, which reflects the remission stage [17]; only received atypical antipsychotics; normal vision; and right-handed. Exclusion criteria: a history of brain trauma, epilepsy and other neurological diseases or serious physical diseases; a history of mental retardation; a history of substance abuse in the past 30 days (except smoking); received electroconvulsive therapy in the past year; a history of using typical antipsychotics; and pregnancy or planning to become pregnant.

Three people in SZs declined because they could not understand the instructions of the MCCB and could not complete it. All SZs and HCs were matched by sex and age.

After describing the study to the subjects, written informed consent was obtained before the study was conducted, and subjects were offered an incentive of Ұ50 per session. The Ethics Committee of the First Affiliated Hospital of the Medical School of Zhejiang University approved the study, which was performed in accordance with the Helsinki Declaration (No. 2018533) and was previously registered in the China Clinical Trial Registry (No. ChiVTR1800016121).

### Virtual reality procedure

VRCTS was designed to assess cognitive function, performing different shopping tasks with different lists. The VRCTS simulated a supermarket with a variety of goods, such as drinks, tea sets, kitchen ware, fruits and vegetables. There was also a shopping cart in the simulation. Unity 5.3.5f1 (https://unity3d.com) and visual studio 2015 (Microsoft) were used to design and create the VRCTS.

The VRCTS included two tasks (task a and task b), and each task consisted of 4 different levels in total. Both tasks required participants to find goods and put them into shopping cart. Task a required participants to find goods of certain type, such as fruits, vegetables and drinks, and task b instructed participants to find specific goods, such as apples, tomatoes and cola. Participants need to finish task a and task b successively, from level 1 to level 4. The number of goods ranged from 3 to 6 as the task level increased from 1 to 4. The working memory (WM) span needed increased with the increasing number of goods. As a result, the different task levels represented different levels of difficulty.

Before each task started, there was a practice task that was used to bring all participants up to their best level of performance, and the outcome of the practice task was not included in statistical analyses. Participants became familiar with the procedures in the practice task as follows:

The participants put on the helmet to begin the VRCTS task;

A list of shopping goods appeared in the VR device, and the participant read the list and closed it after memorization;

The participants chose items accordingly and put them in the shopping cart in the virtual supermarket using joysticks;

If the participant forgot the contents of the list, he or she could press the button on the joysticks, and the list would appear again.

The participant was asked to finish task a and task b successively, using the same procedures as the practice task.

When the participant placed all of the goods from the list into the shopping cart, the computer software automatically recorded the correct number of items, errors, and completion time of each task and calculated the accuracy. The accuracy was equal to the correct number of items divided by the total number of goods in the cart.

If the accuracy was less than 100% the first time, the participant repeated the level. The second accuracy value was statistically analysed.

Completion time and accuracy were the major outcomes of the VR task that were used to evaluate cognitive function. The experiment did not limit the completion time of the task to allow each participant to obtain the highest possible accuracy values.

**Fig. 1:**
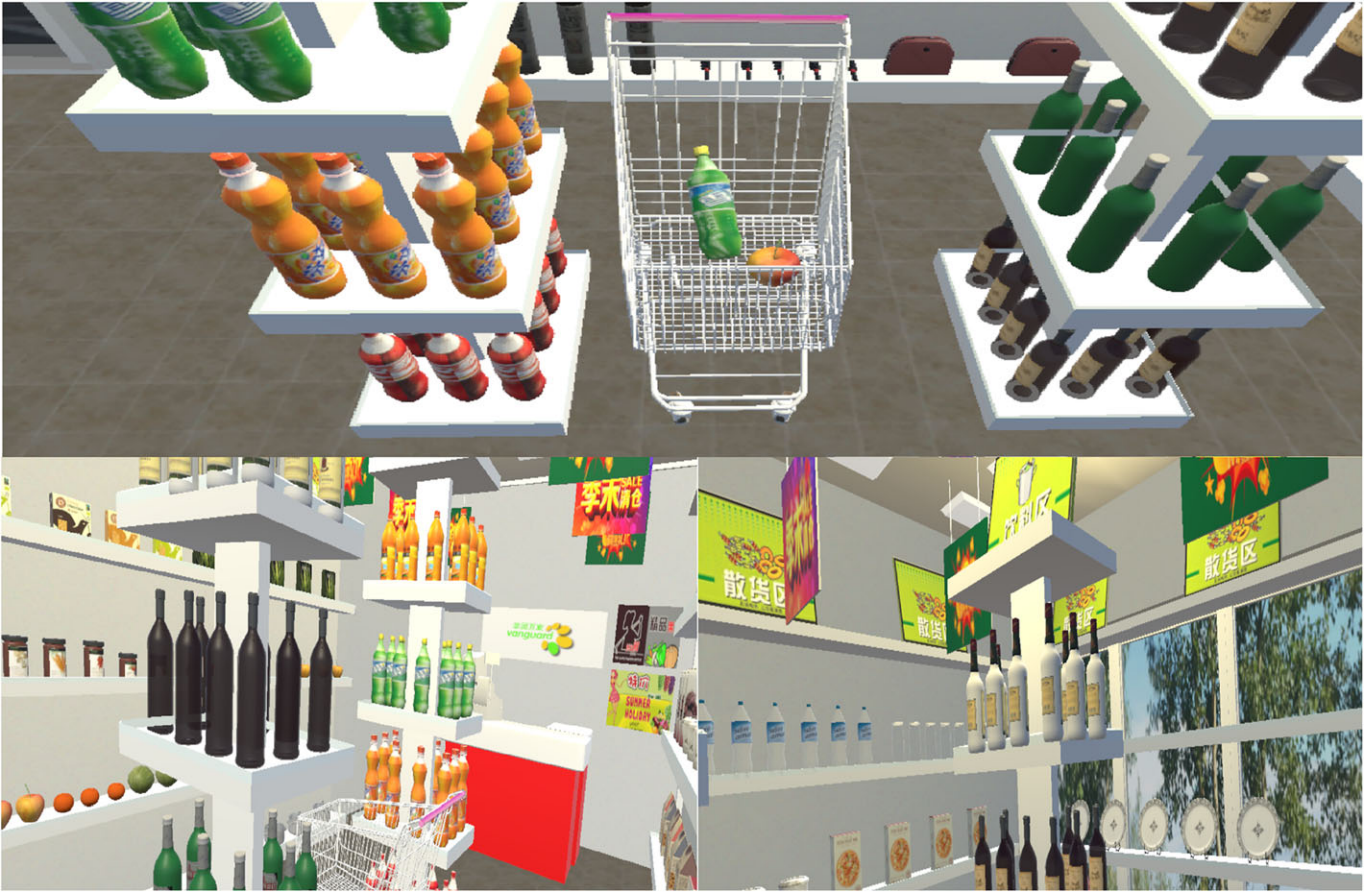
Screenshots showing different views within the virtual reality supermarket developed by our team. The shopping cart (upper) and the goods (lower) are shown

The VR situation was presented as follows (see Fig. 1).

### Cognitive assessment

A trained psychiatrist assessed cognitive function using the MCCB. The MCCB includes 10 neurophysiologic tests that are clustered in 7 cognitive domains: speed of processing (SP), attention/vigilance (AV), WM, verbal learning (VeL), ViL, reasoning/problem solving (RPS), and social cognition (SC) [18]. Each domain score was standardized to a T score using the MCCB computer scoring program (Psychological Assessment Resources, Inc., version 2.1.1). The overall composite T score (CC) was calculated by averaging the standardized value of each test’s T score.

### Auxiliary diagnosis model and verification

We constructed an SVM-based cognitive impairment auxiliary diagnosis model for SZs in the remission stage based on outcomes from 57 subjects (32 SZs and 25 HCs), and outcomes from 18 additional subjects (9 SZs and 9 HCs) were analysed to verify the diagnostic effect.

### Statistical analysis

The data are expressed as the mean ± SD for continuous variables. T-tests were used to compare age and education years of the SZs and HCs. The T scores of the MCCB and outcomes of VRCTS were analysed using covariance analysis (covariates: years of education). Correlation of T scores of the MCCB and outcomes of VRCTS was analysed using Pearson correlation. Statistical analyses were performed using SPSS version 19.0 (IBM, Chicago, IL, USA) for Windows. For verification of the diagnostic model, DTREG (https://www.dtreg.com/) was used to test the diagnostic model.

## Results

### Characteristics of SZs and HCs

We recruited 35 SZs in the remission stage and 25 HCs. Data from 3 of the 35 SZs were excluded because the participants could not finish the MCCB. 32 SZs and 25 HCs completed the VRCTS and the MCCB. 5 SZs and 4 HCs felt dizzy during the VR tasks, but they finished all tasks because the dizziness was tolerable. No other uncomfortable feelings were reported. The age of SZs ranged from 24 to 54 years, and the age of HCs ranged from 28 to 51 years (mean age = 38.84 years SD=5.56). Subject demographic and clinical characteristics are provided in Table 1. The disease duration of SZs ranged from 12 to 384 months. SZs received second-generation antipsychotics for treatment. There were no significant differences in age or sex between groups (all Ps > 0.05). However, SZs had fewer years of education than HCs (see Table 1).

**Table 1:** demographic and clinical characteristics of schizophrenic patients and healthy controls

group	SZ (*n*=32)	HC (*n*=25)	t/χ^2^	*p*
Age (years)	42.69±9.01	38.84±5.56	1.873^a^	0.066
Gender (male/female)	16/16	8/17	1.865^b^	0.172
Education (years)	10.50±3.14	16.28±1.72	−8.270^a^	< 0.001**
Age of first onset (year)	24.63±6.89			
Duration (months)	217.88±108.11			

**note:**^a^ t-test; ^b^ χ^2^ test; *P< 0.05, **P< 0.001

**Abbreviations:** *SZ* patients with schizophrenia, *HC* health controls

### Cognitive function of SZs and HCs

It took each participant approximately 1 h to finish the MCCB assessment. Covariance analysis (education) showed that SZs were significantly impaired in SP, AV, VeL, ViL, RPS, and SC compared to HCs (all Ps < 0.003) (see Table 2).

**Table 2:** MCCB results of schizophrenic patients and healthy controls

Category	SZ(*n=*32)	HC(*n=*25)	F	*p*
SP	23.38±11.22	52.38±8.58	57.970	< 0.001**
AV	31.53±8.60	51.90±7.90	23.262	< 0.001**
WM	41.94±12.11	51.38±18.72	0.312	0.579
VeL	32.63±6.54	46.67±8.15	9.680	0.003*
ViL	32.69±11.71	54.90±8.62	19.584	< 0.001**
RPS	35.84±5.46	49.90±10.67	23.233	< 0.001**
SC	21.50±7.25	43.50±10.02	26.096	< 0.001**
CC	31.38±5.21	50.14±6.37	49.788	< 0.001**

**Note:** *P< 0.05, **P< 0.001

**Abbreviations:** *SZ* patients with schizophrenia, *HC* health controls, *SP* speed of processing, *AV* attention-vigilance, *WM* working memory, *VeL* verbal learning, *ViL* visual learning, *RPS* reasoning/problem solving, *SC* social cognition, *CC*Composite T score

### Comparison of performance on the VRCTS between SZs and HCs

The average total time taken to complete the VRCTS in SZs (1061±427 s) was significantly longer than the HCs (389±226 s). SZs spent significantly more time completing the different levels compared to HCs in task a level 1, level 2 and level 3 and task b level 2 and level 3 (Bonferroni correction, all Ps < 0.0125). However, there was no differences in accuracy of between SZs and HCs for any task (see Table 3).

**Table 3:** VRCTS outcome of patients with schizophrenia and healthy controls

Tasks		SZ (*n*=32)	HC (*n*=25)	*p*
Task a level 1	Time to complete	88.15±55.30	31.75±26.40	< 0.001**
	Accuracy	0.97±0.10	0.96±0.11	0.296
Task a level 2	Time to complete	117.04±81.57	29.91±11.62	0.008*
	Accuracy	0.95±0.12	0.95±0.10	0.481
Task a level 3	Time to complete	139.81±76.51	37.32±17.62	0.006*
	Accuracy	0.93±0.12	0.95±0.09	0.297
Task a level 4	Time to complete	152.13±121.55	44.10±18.21	0.057
	Accuracy	0.93±0.09	0.96±0.09	0.325
Task b level 1	Time to complete	72.36±78.00	43.26±50.90	0.351
	Accuracy	0.94±0.13	0.89±0.19	0.183
Task b level 2	Time to complete	106.97±60.92	44.21±34.56	0.012*
	Accuracy	0.89±0.17	0.86±0.19	0.825
Task b level 3	Time to complete	132.98±71.84	54.69±41.16	0.010*
	Accuracy	0.88±0.15	0.87±0.11	0.950
Task b level 4	Time to complete	179.83±117.25	95.28±131.59	0.211
	Accuracy	0.90±0.13	0.87±0.16	0.132

**Note**:Bonferroni correction *P< 0.0125,**P< 0.001

**Abbreviations:** *SZ* patients with schizophrenia, *HC* health controls

### Correlations of VRCTS outcome with the MCCB

**Fig. 2:**
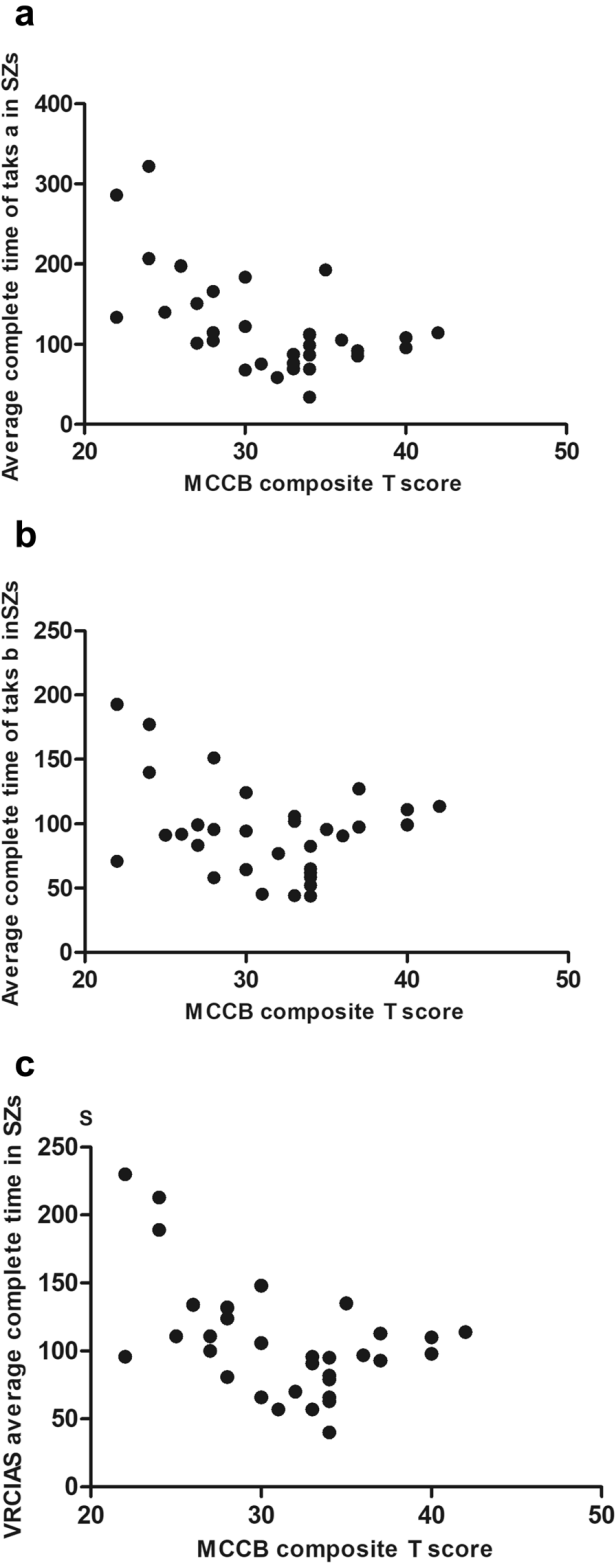
Scatter plots showing the correlations between the completion time of the VRCTS and the MCCB CC in SZs. The completion time of task a and MCCB CC (**a**), the completion time of task b and CC (**b**) and the average completion time of VRCTS and MCCB CC (**c**)

For this analysis, we selected the completion time of each task level and the average completion time and correlated these values with the MCCB CC. These correlations are presented in Table 4 and Fig. 2. The average completion times of task a and task b significantly negatively correlated with the MCCB CC in SZs (Bonferroni correction, *P*< 0.0125). However, there was no similar correlation between the outcomes of the VRCTS and MCCB CC in HCs.

**Table 4:** Pearson correlations between VRCTS and MCCB in SZs and HCs

SZs (*n*=32)			HCs (*n*=25)		
Time to complete	MCCB CC	p	Time to complete	MCCB CC	p
Task a level 1	− 0.16	0.389	Task a level 1	−0.028	0.907
Task a level 2	−0.494	0.005*	Task a level 2	0.23	0.329
Task a level 3	−0.694	< 0.001**	Task a level 3	0.274	0.242
Task a level 4	−0.329	0.071	Task a level 4	−0.223	0.344
Mean of task a	−0.555	0.001*	Mean of task a	0.052	0.826
Task b level 1	−0.387	0.031	Task b level 1	−0.97	0.683
Task b level 2	−0.280	0.128	Task b level 2	0.156	0.523
Task b level 3	−0.232	0.209	Task b level 3	0.316	0.187
Task b level 4	−0.021	0.91	Task b level 4	0.378	0.11
Mean of task b	−0.461	0.009*	Mean of task b	0.124	0.625
Mean of all task	−0.562	0.001**	Mean of all task	0.126	0.618

**Note:**Bonferroni correction *P< 0.0125, **P< 0.001

**Abbreviations:** *SZ* patients with schizophrenia, *HC* health controls, *CC* Composite T score

We also analysed the average completion times of task a and task b with each MCCB domain. The results showed that the outcomes of VRCTS negatively correlated with SP, AV, WM and VeL (all Ps < 0.05). But there is no significant correlation in HCs (see Table 5).

**Table 5:** Pearson Correlation between MCCB domains T scores and VR completion time

MCCB domain	VRCTS	SZs		HCs	
		r	p	r	p
SP	Average of task a	−0.481	0.006*	0.251	0.272
	Average of task b	−0.506	0.004*	0.128	0.601
AV	Average of task a	−0.459	0.009*	−0.058	0.807
	Average of task b	−0.391	0.03*	0.139	0.581
WM	Average of task a	−0.378	0.036*	0.077	0.741
	Average of task b	−0.164	0.378	−0.065	0.790
VeL	Average of task a	−0.439	0.013*	0.048	0.836
	Average of task b	−0.415	0.02*	0.428	0.068
ViL	Average of task a	−0.351	0.053	−0.037	0.873
	Average of task b	−0.243	0.187	0.297	0.217
RPS	Average of task a	−0.315	0.085	−0.118	0.611
	Average of task b	−0.316	0.084	0.062	0.801
SC	Average of task a	−0.102	0.587	−0.002	0.994
	Average of task b	−0.159	0.394	−0.286	0.250

Note: *P< 0.05, **P< 0.001

**Abbreviations:** *SP* speed of processing, *AV* attention-vigilance, *WM* working memory, *VeL* verbal learning, *ViL* visual learning, *RPS* reasoning/problem solving, *SC* social cognition, *SZs* patients with schizophrenia, *HCs* healthy controls

The use of VRCTS as an auxiliary method to identify cognitive impairment.

**Fig. 3:**
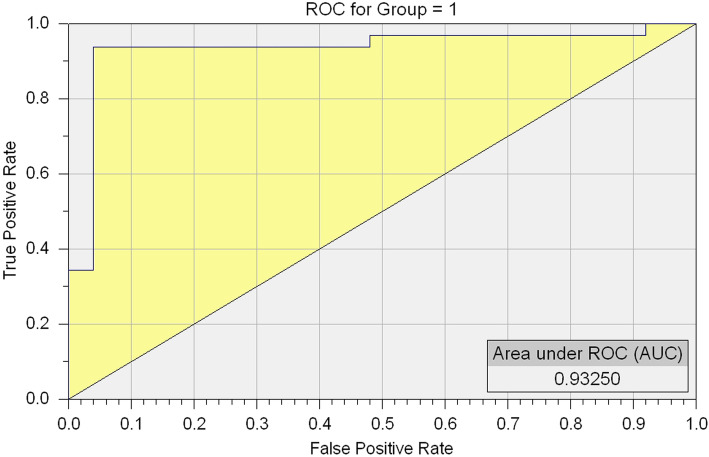
ROC curve distinguishing HCs and SZs.

Our results showed that the cognitive impairment auxiliary diagnosis model correctly classified SZs and HCs based on cognitive impairment. The completion time of VRCTS classified individuals based on cognitive impairment with 92.98% accuracy, 90.63% sensitivity, and 96% specificity. The area under the receiver operating characteristic (ROC) curve was 0.9325 (see Fig. 3). Verification based on another 18 subjects showed 88.89% accuracy, 88.89% sensitivity, 88.89% specificity, 88.89% positive predictive value and 88.89% negative predictive value.

## Discussion

A virtual situation called VRCTS was constructed for the present study, and it simulated a real supermarket to assess cognitive function and examine the validity and safety of VR technology in Han Chinese SZs in the remission stage. SZs required nearly 18 min to complete the VRCTS compared to 6 min for HCs, which is significantly shorter than the time required to complete the MCCB.

The VRCTS was divided into two tasks, and each task consisted of 4 levels that required an increasing WM span. Task a was used to evaluate the processing of information related to certain categories, and task b was used to assess the processing of specific information. We designed different levels for each task, such as task a level 1 and level 2. The number of goods participants needed to find ranged from 3 to 6 as the task level increased. Two variables were used to measure the outcomes: accuracy of performance and the time to complete the task. Because there were no differences in task accuracy between SZs and HCs, it was meaningful to compare the completion times of the two groups.

Our results showed that the completion times for task a and task b of SZs were significantly higher than the completion times of HCs, which was likely due to the cognitive impairment of SZs. These findings are consistent with some studies. Thirty-three SZs and 39 HCs performed 10 trials of a virtual radial arm maze task, and the results showed that SZs took more time to finish the task compared to HCs [19]. Other studies also showed that SZs performed worse than HCs in the Virtual Action Planning-Supermarket (VAP-S) study and a Virtual Reality Functional Skills Assessment (VRFAS) [20, 21]. The use of VR may assess the severity of theory of mind (ToM) impairment in SZs [22].

To finish the task of VRCTS, participants needed to read the list, find the goods on the list and put the goods into a shopping cart. Many cognitive domains are involved in these processes, such as SP, AV, WM and VeL. There were also significant negative correlations between outcome of VRCTS and the MCCB composite score and some domains, like SP, AV, WM and VeL. These correlations support our hypothesis that the VRCTS outcome reflects the cognitive impairment of SZs in the remission stage and primarily reflects SP, AV, WM and VeL.

Our results showed that the VRCTS distinguished SZs in the remission stage and HCs with high accuracy (88.89%), sensitivity (88.89%) and specificity (88.89%). This result means that SZs in the remission stage may be separated from healthy people based on their performance in the VRCTS. This result is consistent with a study of a VR prospective memory test, which demonstrated that the VR test examined prospective memory deficits in SZs with high sensitivity (92.9%) and specificity (75%) [14].

In summary, VR provides participants a feeling of presence that is similar to real life [23, 24]. The present study demonstrated that VR technology, such as the VRCTS, was a time-saving, efficient and attractive method to evaluate cognitive function. Our model constructed a virtual situation that was applicable to Han Chinese people to evaluate cognitive function, and its outcome significantly correlated with the MCCB. The VRCTS helped distinguish SZs in the remission stage and healthy people based on cognitive impairment, which might support its use as a new adjunctive examination to evaluate cognitive impairment in SZs in the remission stage, especially people of Han Chinese descent. Although more research is needed, VR may be an attractive, safe and convenient auxiliary method for assessments in schizophrenics in the future.

This study had some limitations. First, the sample size was small. Second, we did not ask participants to assess their preference for MCCB vs. VRCTS. Therefore, this study lacks feedback from participants after they finished the VRCTS. As a result, it is difficult to rate the satisfaction and pleasure that the participants experienced. The SZs received different types of second-generation antipsychotics, which may interfere with the patients’ cognitive function. As a result, further studies with SZs using a single second-generation antipsychotic are need to exclude the effects of antipsychotics on cognitive function. Third, the two groups were not education matched, and education may influence participants’ performance in a virtual supermarket. Therefore, we used covariance analysis (covariates: years of education) to compare the outcome between SZs and HCs. Furthermore, our conclusion may not applied to first-onset, medication-naïve patients with less obvious cognitive impairment because the patients in our research were all SZs in the remission stage.

## Conclusions

The VRCTS is a highly sensitive measure of the cognitive functions associated with the MCCB test. These results might support the use of VR technology in the assessment of cognitive function in SZs in the remission stage in Han Chinese patients.

## Data Availability

The datasets used and/or analysed during the current study are available from the corresponding author on reasonable request.

## Abbreviations

VR: Virtual reality
MCCB: MATRICS Consensus Cognitive Battery
SZs: Patients with schizophrenia
HCs: Healthy controls
VRCTS: VR cognition training system
CC: Composite T score
WM: Working memory
ViL: Visual learning
VRNT: VR navigation task
SVM: Support vector machine
SP: Speed of processing
AV: Attention/vigilance
VeL: Verbal learning
RPS: Reasoning/problem solving
SC: Social cognition
VAP-S: Virtual Action Planning-Supermarket
VRFAS: Virtual Reality Functional Skills Assessment
